# Teaching nursing skills without detailed protocols: effects of an implicit learning strategy in nursing education

**DOI:** 10.1007/s10459-025-10421-y

**Published:** 2025-03-25

**Authors:** Natasja Van Brakel-Van Lobenstein, Saskia T. Van Leeuwen-Prins, Loes Verdoes, G. Mariëlle De Waal, Maaike C. J. Kamsteeg, Raôul R. D. Oudejans, Jeannie Devereaux, Yvonne van Zaalen, Jeroen Dikken, Peter G. Renden

**Affiliations:** 1 Department of Dermal Therapy, Faculty of Health, Nutrition and Sport, The Hague University of Applied Sciences, The Hague, The Netherlands; 2 Research Group Relational Care, Centre of Expertise Health Innovation, The Hague University of Applied Sciences, The Hague, The Netherlands; 3 Department of Nursing, Faculty of Health, Nutrition and Sport, The Hague University of Applied Sciences, The Hague, The Netherlands; 4 Department of Human Movement Sciences, Amsterdam Movement Sciences, Vrije Universiteit Amsterdam, Amsterdam, The Netherlands; 5 Centre of Expertise Urban Vitality, Amsterdam University of Applied Sciences, Amsterdam, The Netherlands; 6 Institute for Health and Sport, Victoria University, Melbourne, VIC Australia; 7 Research Group Urban Ageing, Centre of Expertise Governance of Urban Transitions, The Hague University of Applied Sciences, The Hague, The Netherlands; 8 Research Group Rehabilitation and Technology, Centre of Expertise Health Innovation, The Hague University of Applied Sciences, The Hague, The Netherlands; 9 Department of Educational Development and Research, Faculty of Health, Medicine and Life Sciences, School of Health Professions Education, Maastricht University, Maastricht, The Netherlands

**Keywords:** Health care education, Instruction, Skill learning, Transfer, Motor learning, Working memory

## Abstract

There is a wide range of literature suggesting that implicit learning is more effective than explicit learning when acquiring motor skills. However, the acquisition of nursing skills in educational settings continues to rely heavily on detailed protocols and explicit instructions. This study aimed to examine the necessity for comprehensive protocols in the acquisition of nursing skills. In the context of bandaging techniques, three studies were conducted to investigate whether students who practiced with an instruction card containing minimal instructions (implicit group) performed comparably to the students who practiced with a protocol containing step-by-step instructions (explicit group). Study 1 was designed to determine whether both groups performed equally well in applying a bandage during training. Study 2 and 3 were designed to determine if both groups performed equally well during a retention and transfer (multitasking) test, administered after a series of three training sessions. In comparison with the explicit group, the implicit group demonstrated comparable performance with their practice attempts in Study 1 and performed equally well during the retention and transfer test in Study 2. Furthermore, several results from Study 3 indicated better performance of the implicit group. In conclusion, the use of protocols with explicit step-by-step instructions may not be essential for the acquisition of nursing skills. Instead, instructional methods that facilitate implicit learning may be preferable, as students in the implicit group demonstrated at least comparable performance in all studies and tended towards greater consistency when multitasking.

**Supplementary Information:**

The online version contains supplementary material available at 10.1007/s10459-025-10421-y.

## Introduction

In the field of nursing education, effectively integrating knowledge, skills, and attitudes, and transferring classroom learning into clinical practice present significant challenges (Van Merriënboer et al., 2025). Theoretical frameworks are crucial as they provide student nurses with a foundation of knowledge; however, the application of this knowledge in practical settings is where the complexity of nursing truly emerges (Bjørk et al., 2013; Christensen, 2011; Dikken et al., 2018; Günay & Kılınç, 2018)*.* Integration is vital, as this is most apparent when theoretical knowledge is applied to practical, patient-centered scenarios (Chernikova et al., 2020; Crowe et al., 2018; Hawkins et al., 2019). A poignant example of this is the task of bandaging, which may appear straightforward but varies considerably based on the patient’s physical characteristics. In practical terms, bandaging a patient with higher adiposity levels may require multiple layers of bandages and additional padding to achieve uniform pressure distribution, differing from the approach suitable for patients with lower adiposity levels. This example illustrates the necessity for nurses to adapt basic procedures to the specific needs of each patient, often while managing simultaneous tasks such as patient communication and symptom monitoring (Renden & Dikken, 2023).

The current teaching of nursing skills follows a traditional pedagogy that heavily relies on detailed protocols and explicit instructions (Renden & Dikken, 2023). Such an approach assumes a straightforward progression in skill acquisition, where students are expected to reproduce specific ideal movements out of context, often focusing on memorizing step-by-step procedures. This method, which has been described in the literature as explicit learning, is structured and systematic (Kleynen et al., 2014). However, it may not adequately prepare students for the unpredictable and multifaceted nature of clinical practice, where the ability to adapt and respond to dynamic situations is essential (Van Merriënboer et al., 2025). Considering that explicit learning is posited to rely heavily on verbal working memory (Kleynen et al., 2014), this approach may prove disadvantageous as it diminishes the cognitive resources available for simultaneous tasks, such as patient communication, clinical reasoning, and operating under time constraints (Davids et al., 2008; Masters et al., 2019). Given the attentionally demanding and complex characteristics of nursing, it is worth investigating whether explicit learning is still preferable for nursing education programs, or whether these programs should focus more on available alternatives.

Explicit learning is associated with detail-oriented instructional methods and presumes that motor learning necessarily starts with declarative knowledge about the skill (Davids et al., 2008; Fitts & Posner, 1967)*.* In the context of bandaging, students are provided with a detailed protocol comprising multiple pages of instructions on the technique. Following this, the students receive a demonstration in which the teacher verbally reiterates most of the instructions. Subsequently, the students commence practising in accordance with the instructions delineated in the protocol. Scientific studies over the past decades have shown that it is also possible to learn implicitly (Masters et al., 2019). With implicit learning, students increase motor skills without (or with reduced) use of declarative knowledge (Kleynen et al., 2014). An example of instructional methods that encourage implicit learning is the use of metaphors (analogy learning). Appropriate metaphors encapsulate the overarching structure of the skill, thus minimising the need for conscious processing by the learner. For example, in basketball free throws, one might use the analogy: ‘Shoot as if you are trying to place cookies into a cookie jar on a high shelf’ (Lam et al., 2009). Teaching by analogy reduces the number of instructions (and the cognitive load) for students while practicing (Kal et al., 2018; Kleynen et al., 2014, 2015).

Numerous experimental studies outside the nursing context, particularly in sports, have demonstrated that the performance of explicitly learned skills tends to be less robust in conditions that demand high levels of attention, compared to implicitly learned skills (Button et al., 2020; Liao & Masters, 2001; Lohse et al., 2010; Masters et al., 2019; van Duijn et al., 2019). Implicit learning makes less use of declarative knowledge and therefore relies less on students’ working memory, leaving more cognitive space for multitasking (Davids et al., 2008; Masters et al., 2008a). Furthermore, it provides students with the opportunity to perform skills in various ways, which is essential for learning to adapt their actions to the unique characteristics of each patient (Renden & Dikken, 2023; Taraporewalla et al., 2023, 2024). These benefits could facilitate the transfer of the learned skills from the educational setting to clinical practice (Beckers et al., 2005; Kahol et al., 2011; Marton, 2006; Masters et al., 2008a).

Given the assumed limitations of explicit learning (Masters et al. 2008a, 2008b, 2010; Poolton et al., 2007) and the demanding and complex nature of nursing, it is worth reconsidering the prevailing approach in nursing education. Particularly, the cognitive load associated with explicit learning appears to be a disadvantage when later integrating technical skills with other tasks such as communicating and clinical reasoning. Instead, it would seem beneficial to facilitate implicit learning in educational nursing programs (Masters et al., 2008a). Several encouraging studies demonstrate the application of this approach in the education of healthcare professionals (Beckers et al., 2005, 2007; El-Kishawi et al., 2023; Masters et al., 2008a; Poolton et al., 2016). Prior to implementing education strategies that facilitate implicit learning in nursing education, several issues require investigation. One such issue is whether the results observed in a controlled experimental setting are transferable to the reality of an educational course. Additionally, the skills tested in most implicit learning experiments, such as executing a table tennis forehand, involve fewer sequences of actions compared to many nursing skills like bandaging. Considering the complex sequence of actions required in nursing, it may be essential to provide more detailed instructions, particularly for novice students.

The primary objective of this research was to evaluate whether instructional methods that facilitate implicit learning are as effective as explicit learning in the acquisition of nursing skills. The research was conducted through three separate studies. Study 1 examined how well and in what time novice students performed two practice attempts of a bandaging technique during training, while they received minimal instructions (implicit group) and compared their performance to that of students receiving a training with detailed instructions (explicit group). Studies 2 and 3 further examined the proficiency of novice students in bandaging following three skills training sessions, either in the implicit or explicit group. Following the three training sessions, students completed a retention and a transfer test (Masters et al., 2008a). A retention test requires participants to perform the skill they learned without support from instructors. A transfer test assesses the stability of performance within different environmental demands or a slightly different task (Huang & Mercer, 2001; Masters et al., 2008a). The findings from these studies will contribute to enhancing the methodologies used in skill training, ultimately improving the transfer of learned skills from educational settings to clinical practice.

## Study 1

### Methods

Study 1 was designed to determine if novice nursing students were equally effective at applying a bandage during training with minimal instructions (implicit group) or detailed instructions (explicit group).

### Participants

A total of 189 novice nursing students from the 2020–2021 full-time bachelor’s Nursing cohort were invited to participate in this study, of which 42 volunteered (9 male and 33 female). The age of students ranged from 16 to 34 years, with a mean ± standard deviation age of 20 ± 4 years. All students were novices with bandaging. All students provided informed consent prior to their involvement in the study.

### Task and materials

During the training session, clinician educators (Registered Nurse or Dermal Therapist) taught students to cut and fit the stockinette bandage liner around the lower leg of a peer, incorporating padding materials into the cavities around the feet and legs to prevent skin damage and ensure uniform pressure distribution of the bandage. Students were then taught to apply two short-stretch bandages to the lower leg.

A skills classroom was divided into several workplace stations. Each student had a table, chair, protocol or instruction card, and bandage equipment (stockinette bandage liner, padding, two short-stretch bandages (Jobst Emmerich am Rhein, Germany), tape, and a scissor). The classroom is equipped with a Noldus Viso camera system (AXIS M5525, Wageningen, The Netherlands)*.*

#### Explicit group instructional material

**Fig. 1:**
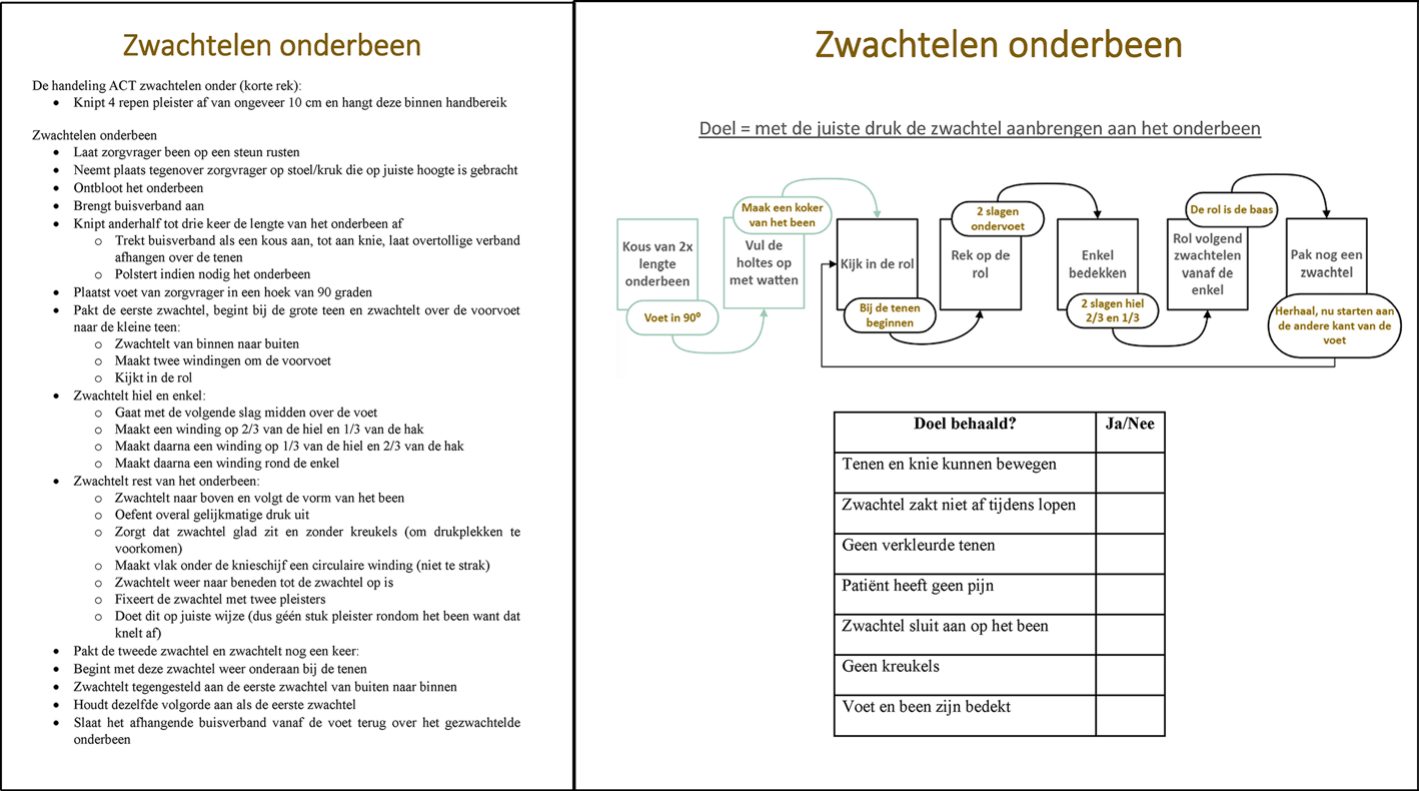
Protocol and instruction card (for details, see Appendix 1 and 2)

Students in the explicit group received a detailed protocol on paper comprising step-by-step instructions on how to perform the technique (Fig. 1, Appendix 1). Besides receiving the instructions on paper, the students also received a demonstration in which the teacher verbally reiterated most of the instructions. These instructions were based on the protocol that is used in clinical care but are a stripped-down version that solely focused on the actions related to bandaging (instructions such as washing hands were omitted) (Bohn Stafleu van Loghum, 2023).

#### Implicit group instructional material

Students in the implicit group received minimal instructions on paper, herein referred to as the instruction card. To develop this instruction card, a task analysis with teachers, researchers, and experts was performed. First, the goals of applying a bandage were determined, to establish when the skill is mastered*.* Then, essential success factors to achieve these goals were identified. Finally, with this information, the instruction card with minimal instructions and implicit strategies was made. By focusing on the essential instructions, the text was reduced from 308 words with explicit step-by-step instructions to an instruction card with a schematic overview of 67 words (Fig. 1, Appendix 2). Besides receiving the instruction card on paper, the students also received a demonstration in which the teacher verbally reiterated most of the instructions of the instruction card.

### Procedure

**Fig. 2:**
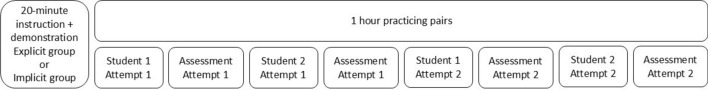
Bandaging training session

The students were randomly divided into an explicit group (*n* = 19) and an implicit group (*n* = 23). Due to the availability constraints of the students, there is a slight difference in the number of students per group (it took more than one session to collect the data: two implicit sessions and two explicit sessions). Ultimately, an available student assistant helped ensure an even number of students (and acted as one of the students), but this person was not part of the study. Each session was conducted in a skills classroom with a trained teacher and researchers present. Following a 20-min instructional period, which also included the demonstration, students engaged in an hour of paired practice in bandaging techniques (Fig. 2).

During the training session, students were required to achieve optimal bandage tension and pressure on the lower leg. Following each bandaging attempt, the instructor evaluated the quality of the application and provided verbal feedback. Time and additional behavioural variables were analysed later, based on the video recordings. Subsequently, students alternated roles. With 5 min remaining, the researcher signalled the conclusion of the training session. Each students was able to complete two attempts of bandaging. Students were encouraged to consult the protocol or instruction card for guidance when questions arose. To determine whether minimal instructions facilitated implicit learning (and step-by-step instructions facilitated explicit learning), students were asked to report the movement-related rules they recalled regarding the technique, following the training session.

### Dependent variables

#### Recall

To analyse the effect of the instructional method, students were asked to recall the movement-related rules about bandaging on paper.

#### Performance

Optimally, bandage pressure would have been directly measured to evaluate performance; however, during Study 1, a sufficient number of pressure-measuring devices were not available per session. Alternatively, performance was assessed based on the objectives outlined in the protocol and instruction card. To assess performance, the following criteria were rated from 1 (wrong) to 5 (correct) by the trained teacher:

- Foot and leg are covered
- Bandage fits tightly around the leg
- No wrinkles
- Patient has no pain
- No discoloured toes
- Toes and knee can move
- Bandage does not slip when walking

#### Time

Performance time: the time each students needed per attempt, measured in seconds.

#### Reading instructions

To analyse the extent to which students depended on the provided instructions, each attempt was segmented into three distinct phases: Phase 1: Preparation; Phase 2: Application of the first bandage roll; and Phase 3: Application of the second bandage roll. The following criteria were analysed:

- Frequency of looking at instructions: how often does the student look at the protocol/instruction card?
- Time looking at instructions: how many seconds does the student look at the protocol/instruction card?

### Data analysis

To determine differences in recalled Movement-related rules between the explicit and implicit group, an Independent Samples *t*-test was performed. Effect sizes were calculated using Cohen’s *d* (Cohen, 1988) with 0.20 or less, about 0.50, and 0.80 or more, representing small, moderate, and large effects, respectively. 2 × 2 mixed Analysis of Variance (ANOVA) tests were conducted to evaluate the variables of Performance, Time and Reading instructions*.* These analyses considered Group (explicit vs. implicit) as a between-subjects factor and Attempt (first attempt vs. second attempt) as a within-subjects factor. The variables regarding Reading Instructions were analysed per phase. The alpha level for significance was set at *p* < 0.05. Effect sizes were calculated using partial eta squared (*η*^2^)*. η*^2^ = 0.01 indicates a small effect, *η*^2^ = 0.06 a medium effect, and *η*^2^ = 0.14 a large effect. All analyses were performed in SPSS 28 (IBM SPSS, IBM Corp, Somers NY).

### Results

For reasons of readability, we present the most important results here. The full data and statistical analyses can be found in the supplementary files.

#### Recall

The Independent Samples *t*-test on movement-related rules indicated no significant difference between groups. This result indicates that students in the explicit group did not remember more movement-related rules than students in the implicit group, even though they had more rules available to them.

#### Performance and time

The results of the Performance and Time variables showed no significant differences between the two groups, suggesting that, on average, both the explicit and implicit group performed comparably well and needed comparable time to complete an attempt. Of the Performance and Time variables, there were two significant differences between attempt 1 and 2. students in both groups exhibited improved performance on the variable “no wrinkles” in attempt 2 compared to attempt 1 (*p* < 0.001, *η*^2^ = 0.65). Furthermore, students in both groups completed attempt 2 faster than attempt 1 (*p* < 0.001, *η*^2^ = 0.36). There were no significant interaction effects between group and attempt.

#### Reading instructions

The ANOVAs on Frequency of looking at instructions showed no significant differences per group (Table 1), but did show significant differences per attempt 1 and 2 in all three phases (P1: *p* < 0.001, *η*^2^ = 0.48, P2: *p* < 0.001, *η*^2^ = 0.36, P3: *p* < 0.05, *η*^2^ = 0.13). Students looked less frequent at their instruction in the second attempt. Furthermore, there was a significant interaction between group and attempt in Phase 1 (*p* < 0.05, *η*^2^ = 0.12). Students in the explicit group looked more frequent to their instructions in the first phase of their first attempt than students in the implicit group (*M* explicit = 3.78, *SD* = 4.11; *M* implicit = 1.78, *SD* = 1.91, *p* < 0.05, *d* = 0.69), but there was no difference between groups in the first phase of the second attempt (*M* explicit = 0.22 *SD* = 0.73; *M* implicit = 0.17, *SD* = 0.49, *p* = *0.80*).

**Table 1:** Mean ± standard deviation of variables concerning Reading instructions

	Explicit		Implicit	
	Attempt 1	Attempt 2	Attempt 1	Attempt 2
Frequency looking at instructions Phase 1	3.78 (4.11)	.22 (.73)	1.78 (1.91)	.17 (.49)
Frequency looking at instructions Phase 2	2.67 (3.68)	.22 (.73)	1.48 (1.38)	.13 (.34)
Frequency looking at instructions Phase 3	1.17 (2.62)	.00 (.00)	.22 (.52)	.04 (.21)
Time looking at instructions Phase 1 (seconds)	34.06 (38.78)	1.22 (4.71)	4.43 (6.17)	.39 (1.12)
Time looking at instructions Phase 2 (seconds)	19.56 (24.84)	1.56 (4.97)	4.35 (5.85)	.43 (.99)
Time looking at instructions Phase 3 (seconds)	5.94 (12.89)	.00 (.00)	.26 (.62)	.04 (.21)

The ANOVAs on Time looking at instructions showed significant differences between groups in all three phases (P1: *p* < 0.5, *η*^2^ = 0.24, P2: *p* < 0.05, *η*^2^ = 0.17, P3: *p* < 0.05, *η*^2^ = 0.10), demonstrating that students in the explicit group consulted the protocol longer than the students in de implicit group. Furthermore, there were significant differences between attempt 1 and 2 in all three phases (P1: *p* < 0.001, *η*^2^ = 0.358, P2: *p* < 0.001, *η*^2^ = 0.319. P3: *p* < 0.05, *η*^2^ = 0.119, showing that students spent less time reading their instructions in attempt 2 compared to attempt 1. There were also significant interactions between group and attempt in all three phases (P1: *p* < 0.001, *η*^2^ = 0.25, P2: *p* < 0.05, *η*^2^ = 0.16, P3: *p* < 0.05, *η*^2^ = 0.11). Students in the explicit group looked more seconds to their instructions in all three phases (P3 was nearly significant) of their first attempt than students in the implicit group (P1: *p* < 0.01, *d* = 1.10, P2: *p* < 0.05, *d* = 0.90, P3: *p* = *0.07, d* = 0.62), but there was no difference between groups in the second attempt (P1: *p* = *0.42, P2: p* = 0.36, P3: *p* = 0.38).

### Interim discussion study 1

The students in the implicit group of Study 1 demonstrated performance outcomes during practice attempts comparable to those of the explicit group. However, the explicit group required significantly more time to read and comprehend their instructions (protocol) than the implicit group (instruction card) in their first attempt. Notably, most students in the explicit group discarded the protocol throughout their second attempts. The findings highlight potential advantages of incorporating implicit approaches into nursing education. Such approaches appear to maximise practice time (by reducing time spent on reading) and may align better with the demands of multitasking. Nevertheless, the findings from Study 1 are constrained to practice behaviour observed in one single session. To build on these results, Study 2 will explore the learning effects after three training sessions.

## Study 2

### Methods

Study 2 was designed to determine if novice nursing students were equally effective at applying a bandage during a retention and transfer tests, following three training sessions with minimal instructions (implicit group) or detailed instructions (explicit group).

### Participants

**Fig. 3:**
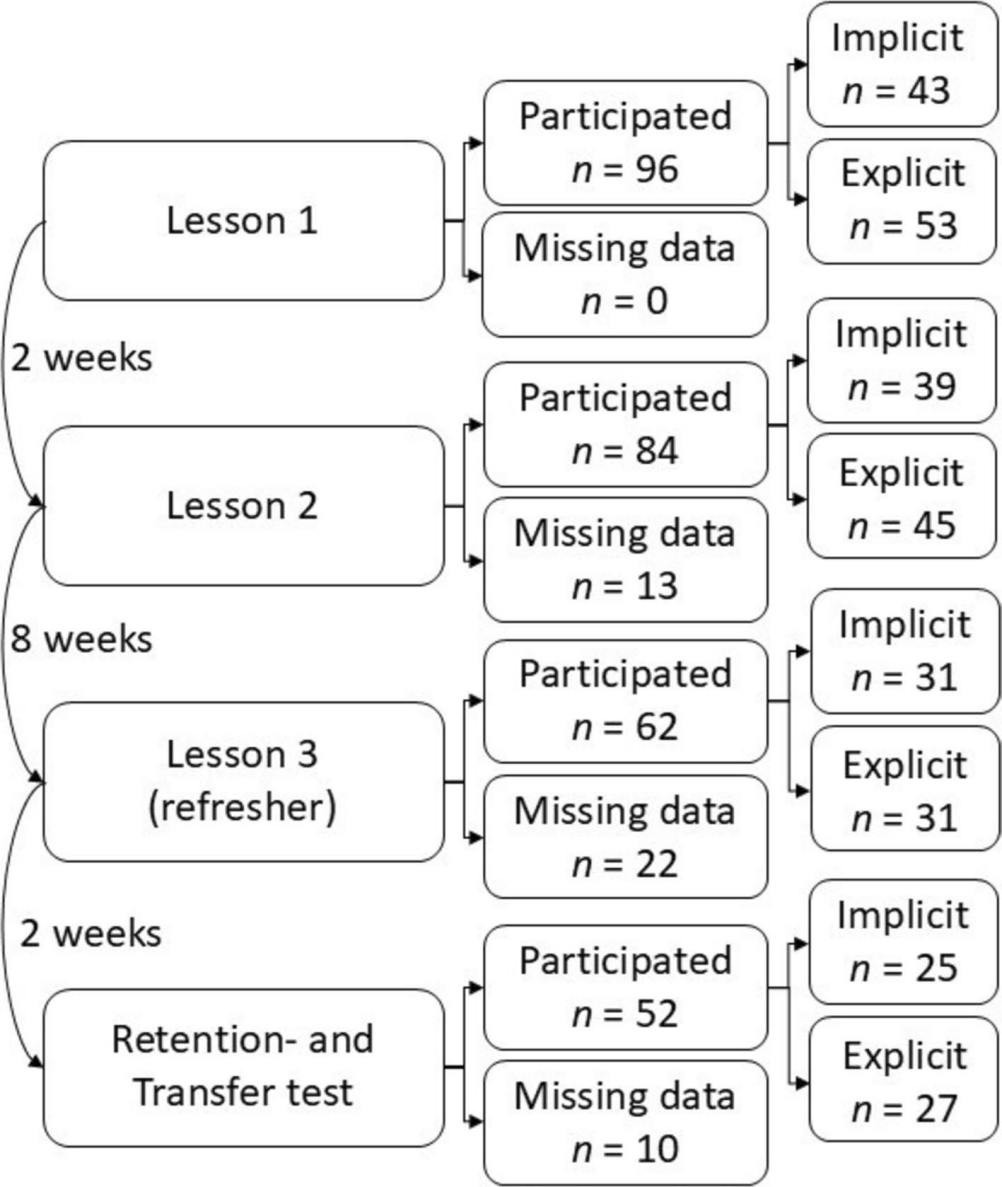
Participants flowchart

This study was conducted among first-year nursing students who were novices with the task of bandaging. All students of cohort 2021–2022 (*n* = 165) were included in Study 2. The non-compulsory nature of the training sessions led to missing data attributable to participant attrition. In total, 52 students (12 male, 40 female) finished the experiment with complete data (Fig. 3). Their age was 21 ± 4 years (mean ± SD) and ranged from 16 to 34 years. All students provided informed consent prior to the study.

### Design and materials

A 2 (group) × 2 (test) design was used. First, students performed three training sessions. Three training sessions is a common number of sessions on bandaging in a Bachelor of Nursing curriculum. The task, practice material and the instructions on paper (step-by-step instructions for the explicit group and minimal instructions for the implicit group) were the same as in Study 1. Also, the content of the training sessions was similar with Study 1. The three sessions started with a demonstration by a trained teacher (RN), followed by practicing bandaging in pairs. Students were allowed to choose the pair themselves and make two attempts of applying a bandage. After each turn, the students called the teacher to receive feedback. After receiving feedback, students switched roles. Two weeks after the last training session, they performed a retention and transfer test.

#### Tests

The retention and transfer test were performed 2 weeks after session 3. The students and a teacher (Registered Nurse or Dermal Therapist) were the only people in the practice room. The student bandaged the leg of the teacher without any demonstration or instruction. After the retention test, students completed a transfer test, in which they were required to bandage a leg of the same teacher while they performed a dual-task. The dual-task was the same as in Masters et al. (2008a). Students were required to simultaneously vocalize random numbers (during the first bandaging roll) and letters (during the second bandaging roll), synchronized with the beeps of an electronic metronome that emitted a sound every second. The metronome and timer were activated as soon as the bandaging material was handled. The teacher monitored the session and intervened if the generation of numbers or letters halted or if it became evident that the sequences were not random.

The tests were recorded with a video camera (Canon Legria HF R706, Netherlands)*.* Each room was equipped with a table, chair, and the necessary bandaging materials, including a stockinette bandage liner, padding, two short-stretch bandages, tape, and scissors. Kikuhime pressure monitoring device (HPM-KH-01, TT Meditrade, Soro, Denmark) was used to measure the pressure at different points under the compression bandage in millimeters of mercury (mmHg) (Brophy-Williams et al., 2017)*.* The Kikuhime pressure measuring device was placed on the legs, using the B1 position (Partsch et al., 2006).

### Procedure

#### Training sessions

The procedure of the training sessions was similar as in Study 1.

#### Retention and transfer test

First, students filled in a form with personal characteristics and signed for informed consent. Next, they performed the retention test, followed by the transfer test. After the student finished a test, the teacher recorded the pressure, measured in mmHg, of both legs in standing position.

In the subsequent class, 1 week after the test (due to time constraints during testing), the students were asked to report the movement-related rules they remembered about the technique.

### Dependent variables

#### Recall

To analyse the effect of the instructional method, students were asked to recall the movement-related rules about bandaging on paper.

#### Performance

To assess the quality of the applied bandage, the pressure (mmHg) was read from the Kikuhime pressure measuring device. While the optimal pressure for reducing chronic extremity swelling remains debated (Partsch et al., 2011), this study utilizes an applied bandage pressure range of 40–60 mmHg based on literature (Damstra et al., 2008; Damstra & Partsch, 2009; Mosti & Cavezzi, 2019; Nederlandse Vereniging voor Dermatologie en Venereologie (NVDV) & Kenniscentrum Wondzorg, 2015; Olszewski, 2008; Partsch et al., 2011)*.* The following variables were derived from the pressure of the applied bandage:

- Pressure of the bandage: measured in mmHg.
- Deviation from the range of 40–60 mmHg: regardless of whether the pressure was below 40 mmHg or above 60 mmHg.
- Pressure between the range 40–60 mmHg: yes or no.
- Consistency pressure: calculated in mmHg by the difference between the retention and transfer test.

#### Time

To analyse the time each students needed per attempt and how that varied between tests, the following variables were measured:

- Performance time: the time each student needed per attempt, measured in seconds.
- Consistency time: the difference between the durations of the retention and transfer test, measured in seconds.

### Data analysis

To determine differences in the variables Movement-related rules, Consistency pressure and Consistency time between the explicit and implicit group, Independent Samples *t*-tests were performed. Effect sizes were calculated using Cohen’s *d* (1988) with 0.20 or less, about 0.50, and 0.80 or more, representing small, moderate, and large effects, respectively. 2 × 2 mixed Analysis of Variance (ANOVA) tests were conducted on the variables “Pressure of the bandage”, “Deviation from the range of 40–60 mmHg”, “Performance time”*.* These analyses considered ‘Group’ (explicit vs. implicit) as a between-subjects factor and ‘Test’ (Retention test vs. Transfer test) as a within-subjects factor. The alpha level for significance was set at *p* < 0.05. Effect sizes were calculated using partial eta squared (*η*^2^)*. η*^2^ = 0.01 indicates a small effect, *η*^2^ = 0.06 a medium effect, and *η*^2^ = 0.14 a large effect. In addition, Chi-square tests were performed on Pressure between 40 and 60 mmHg on the retention test, the transfer test, and on both tests (here also, the level for significance was set at 0.05). All analyses were performed in SPSS 28 (IBM SPSS, IBM Corp, Somers NY).

### Results

For reasons of readability, we present the most important results here. The full data and statistical analyses can be found in the supplementary files. Study 2 showed no significant interactions between *Group* and *Test*. The results are presented based on these two independent variables.

#### Group

The Independent Samples *t*-test on Movement-related rules revealed a significant difference between groups (*p* < 0.05, *d* = 0.78), showing that the implicit group memorized fewer movement-related rules about bandaging, 1 week after the retention and transfer test.

The ANOVAs on the variables Pressure of the bandage, Deviation from the range 40–60 mmHg and Performance time revealed no significant differences between groups. Also, the Independent Samples *t*-test on the variables Consistency pressure and Consistency time and the Chi-Square Tests on the variable Pressure between 40 and 60 mmHg (in all tests) showed no significant differences between groups.

#### Test

There were some significant differences between tests. In both groups, the pressure of the applied bandage was higher in the transfer test than in the retention test (*p* < 0.05, *η*^2^ = 0.80). Furthermore, students in both groups completed the transfer test faster than the retention test (*p* < 0.05, *η*^2^ = 0.13). There were no other significant differences between tests.

### Interim discussion study 2

Study 2 expanded on the findings of Study 1 by assessing the learning effects after three training sessions, extending the analysis beyond practice behaviour in a single session. The implicit group in Study 2 demonstrated performance outcomes comparable to the explicit group in both retention and transfer tests. Additionally, the recall check revealed that students in the explicit group acquired a greater number of movement-related rules compared to the implicit group, indicating a greater reliance on working memory during practice. These findings reinforce the potential benefits of prioritising instructional methods that facilitate implicit learning in nursing education.

Beyond enabling more time for practice (as observed in Study 1), implicit learning appears to support multitasking by reducing demands on working memory capacity. However, across both groups, only a small proportion of students in Study 2 successfully applied the bandage within the desired pressure range in the retention and transfer tests. This suggests that three sessions may not be sufficient to achieve proficiency in bandaging.

It is important to note that the students and teachers in Study 2 were initially accustomed to instructional methods that facilitate explicit learning, with minimal instructions being introduced for the first time. Study 3 will replicate Study 2 but in a context where both students and teachers are more familiar with instructional methods that facilitate implicit learning, potentially yielding further insights.

## Study 3

### Methods

One year following Study 2, teachers and students acquired greater familiarity with the instructional approach of implicit learning as part of a revised skills program. An additional retention and transfer test was implemented to examine how these students of the revised skills program would perform after bandaging training compared to the students of Study 2. Thus, identical to Study 2, Study 3 was designed to determine if novice nursing students were equally effective at applying a bandage during a retention and transfer tests, administered after three training sessions with minimal instructions (implicit group, students from cohort 22–23) or detailed instructions (explicit group, students from cohort 21–22 [Explicit group Study 2]).

### Participants

These additional measurements were collected from first-year nursing students who were novices with the task bandaging. A simple random sample from cohort 22–23 was approached (*n* = 27). All 27 students (3 male, 24 female) participated in the retention and transfer rest. Their age (mean ± SD) was 22 ± 5 years and ranged from 17 to 40 years. All students gave their informed consent before they participated in the study.

Design and materials, Procedure, Dependent variables and Data analysis were the same as in Study 2.

### Results

For reasons of readability, we present the most important results here. The full data and statistical analyses can be found in the supplementary files. Study 3 showed no significant interactions between *Group* and *Test*. The results are presented based on these two independent variables.

#### Group

The Independent Samples *t*-test on movement-related rules revealed a significant difference between groups (*p* < 0.05, *d* = 0.80), showing that the implicit group memorized fewer movement-related rules about bandaging, 1 week after the retention and transfer test.

The ANOVAs on the variables Pressure of the bandage, Deviation from the range 40–60 mmHg, and Performance time revealed no significant differences between groups. The Independent Samples *t*-test analysis on Consistency pressure revealed a significant difference between groups (*p* < 0.05, *d* = 0.58). The average difference between the retention test and the transfer test was larger for the explicit group than for the implicit group, implying that the implicit group was more consistent in their applied pressure than the explicit group. The Independent Samples *t*-test analysis on Consistency time revealed no significant difference between groups.

The Chi-Square Test analysis on Pressure between 40 and 60 mmHg showed no significant difference between the groups in the retention test, but did show a significant difference for the transfer test (*p* < 0.05). In the explicit group, 11 out of 27 students (41%) were able to apply the bandage with a pressure of the between 40 and 60 mmHg. In the implicit group, 19 out of 27 students (70%) were able to apply the bandage with a pressure between 40–60 mmHg. Furthermore, the Chi-Square Test for both tests showed a significant difference between groups (*p* < 0.05). In the explicit group, 6 out of 27 students (22%) were able to apply the bandage with a pressure between 40 and 60 mmHg in both the retention and the transfer test. In the implicit group, 15 out of 27 students (56%) were able to apply the bandage with a pressure between 40 and 60 mmHg in both the retention and the transfer test.

#### Test

The ANOVA on the variable Pressure of the bandage showed a significant difference between tests (*p* < 0.05, *η*^2^ = 0.08), showing that, on average, in both groups, the pressure of the applied bandage was higher in the retention test than in the transfer test. Furthermore, students in both groups completed the transfer test faster than the retention test (*p* < 0.001, *η*^2^ = 0.19). The ANOVA on Deviation from range 40–60 mmHg showed no significant difference between tests.

### Interim discussion study 3

Study 3 expanded on the findings of Study 2 by exploring the transition from initial exposure to instructional methods that facilitate implicit learning to a scenario where students and teachers had become more familiar with this approach. The performance of the implicit group in Study 3 was compared with that of the explicit group in Study 2. Notably, a larger proportion of students in the implicit group of Study 3 successfully applied the bandage within the pressure range of 40–60 mmHg during the transfer test and across both tests overall, compared to the explicit group. Additionally, the implicit group in Study 3 demonstrated greater consistency in maintaining the applied pressure across retention and transfer tests.

The differences between the performance results of Study 2 and Study 3 suggest that effectively integrating instructional methods that facilitate implicit learning into educational programs requires significant time and adaptation. However, as both students and teachers became more accustomed to implicit learning, its potential benefits, especially in contexts requiring multitasking, became evident.

Overall, the findings of Study 3 addressed two key challenges outlined in the introduction. First, they demonstrated that results achieved in controlled experimental settings can be successfully translated to the more dynamic environment of an educational course. Second, they confirmed that complex nursing skills can be effectively taught with minimal instructions, even for tasks involving a series of intricate actions.

## Discussion

The objective of this study was to examine the need for comprehensive protocols in the acquisition of nursing skills, specifically focusing on bandaging techniques. Three studies were conducted to investigate whether students who practiced with an instruction card with minimal instructions (implicit group) performed comparably to the students who practiced with a traditional protocol with step-by-step instructions (explicit group). The results of the three studies suggest that the use of detailed protocols is not necessary for learning a nursing skill. In comparison with the students in the explicit group, the students in the implicit group demonstrated comparable performance with their practice attempts (Study 1) and performed equally well during the retention and transfer test (Study 2). Furthermore, the results of Study 3 may imply that the use of an instruction card with minimal instructions has advantages over the use of a detailed protocol. In the implicit group, a greater number of students were able to apply the bandage within the ideal range of 40–60 mmHg in the transfer test and in total across both tests, in comparison to the explicit group. Furthermore, the results of Study 3 indicate that students in the implicit group were more consistent in applied pressure across the retention and the transfer test than students in the explicit group.

Several research projects on implicit and explicit learning (Cabral et al., 2022; El-Kishawi et al., 2023; Masters et al., 2008a, 2008b, 2010, 2019; Poolton et al., 2007, 2016) have reinforced our view that single-task exercises in nursing education (such as solely practicing a technical skill) should be taught in such a way that the learned skills can be easily applied simultaneously with other learned skills. From that perspective, we argued earlier that explicit leaning may be less suitable for skill learning in nursing education, because it strongly relies on the (verbal) working memory. That may be a disadvantage as students have less cognitive space available for additional tasks (Masters et al., 2019). The results of our study indeed imply that explicit learning methods, such as using an extensive protocol, appear to place a greater demand on students’ verbal working memory. The observations in Study 1 showed that students in the explicit group required significantly more time to read and comprehend the protocol than students in the implicit group. Nevertheless, the students appeared to be unable to recall most of the rules after a single training session. It is possible that the number of rules was excessive, as most students in the explicit group discarded the protocol throughout their attempts. It appeared that students required several sessions to recall the majority of the protocol. This was evidenced by the manipulation checks conducted after Study 2 and 3, which demonstrated that students in de explicit group acquired a greater number of movement-related rules than those in the implicit group. Given that extensive protocols did not yield any benefits in terms of practice and learning in our three studies, it seems beneficial to invest in instructional methods that facilitate implicit learning for nursing education. It allows for more time to practice (less time to read) and seems more congruent with multitasking.

### Implications of this study

The results of this study have a number of implications. To implement more implicit learning in nursing education (and other health care education), it would be beneficial to ascertain further information on the various forms of implicit learning (see for example Kleynen et al., 2014). In this discussion, we will examine the three forms of implicit learning that we used in this experimental study. An often-used application of implicit learning is using an external focus of attention, whereby students focus on the effect of the movement in the environment rather than the movement itself (Lohse et al., 2010). In the instruction card, for instance, the instruction “cover the ankle” (after students made two turns around the forefoot of the patient) was used as an alternative to providing step-by-step instructions for the application of the bandage around the heel and ankle. By providing an instruction that describes the effect of the movement, students were able to assess how they could achieve this effect. Another form we used was analogy learning. To illustrate, during the demonstration, the following metaphorical instruction was used: “apply padding material so that the leg resembles the shape of a tube” (the shorter version used in the instruction card was: “create a tube”). The exercise employed in the transfer test (students simultaneously vocalised random numbers and letters in synchrony with the beeps of an electronic metronome) is a form of dual-task learning. This secondary task engages the working memory, thereby limiting the individual’s ability to consciously control their movements (Masters et al., 2008a).

As outlined in the method section, a series of steps were undertaken which resulted in the production of the instruction card. We first performed a task analysis with teachers, researchers, and experts. Then, the goals of applying a bandage were determined, in order to ascertain the criteria for proficiency. Subsequently, the essential success factors required to achieve these goals were identified. An important success factor was identified as the padding of bony prominences (such as the malleolus and tibia) to achieve an even resting pressure of the bandage. Ultimately, based on this information, potential instructions were formulated, such as “apply padding material so that the leg resembles the shape of a tube”. The systematic approach and collective brainstorming sessions proved beneficial in terms of identifying the necessity of instructions and jointly creating possible instructions. Later, after the completion of this study, we initiated the incorporation of students on a more regular basis. For instance, several students continued to experience difficulties in bandaging the heel and ankle effectively. In collaboration with the students, we devised a strategy to include two illustrative examples of the desired bandage appearance. This helped students to focus their attention externally during practice. Our key recommendation based on this experience is to maintain a process of continuous evaluation and adaptation, ideally in conjunction with students (Bovill et al., 2011; Brooman et al., 2014).

We should also discuss the many non-significant differences between the implicit and explicit groups in this study. Our findings may be in line with conclusions of recent reviews that suggest that the advantages of learning skills implicitly may be moderately sized and need further exploration with larger sample sizes to estimate the effects more accurately (Cabral et al., 2022; Kal et al., 2018)*.* Thus although we clearly advocate for the application of instructional methods that facilitate implicit learning, we want to acknowledge that there is also a time and place for explicit learning (Hutter et al., 2023)*.* Explicit and implicit learning are often presented as two opposing ends of a continuum (Kok, 2023)*,* but the use of instructions in real-life settings is probably less black and white. In addition, the findings from Study 2 and 3 suggest that the integration of implicit learning requires a considerable amount of time. It is our experience in this study that teachers need time to understand the background and to practice with the methods. In Study 2, the teachers and students utilized implicit instructions for the first time. Although the minimal instructions appeared to be as effective as the explicit instructions, there was no significant difference between the two groups. One year later, when teachers acquired greater familiarity with implicit learning and students from that cohort became accustomed to this instructional approach in skills training, Study 3 revealed distinctions between the implicit group of that year and the explicit group of Study 2 from the preceding year*.*

Finally, the results indicate that three training sessions in bandaging are insufficient to become skilful, which could explain the not many significant differences between the groups. The results of Study 2 show that only a small number of students were able to apply the bandage within the desired range of pressure across both tests. Yet, this number improved in Study 3, indicating that implicit learning may had a positive effect. Nevertheless, the results also indicate that students require more sessions. When compared with, for example, a table tennis forehand (Liao & Masters, 2001) or a suturing and knot-tying task (Masters et al., 2008a), students in this study were only able to perform two practice attempts per session. The skills in previous studies had fewer sequences of actions, making it possible to perform more repetitions within a given practice time. Given that in the Netherlands it is common practice to schedule only two or three sessions on bandaging among other skills, we propose the scheduling of more practice time with a large variation of exercises (e.g., Renden & Dikken, 2023) for technical skills in nursing education programs (see also Ericsson, 2014)*.*

### Strengths and limitations

One of the strengths of this study is that we were able to conduct Study 2 and 3 during regular nursing training (Study 1 was organized apart from the regular program), which enhances the representativeness of the data in a typical classroom setting. Furthermore, our findings are based on data from multiple studies conducted over an extended period. This approach allows us to gain insights into the typical experiences of nursing students following a regular Dutch curriculum. In addition, the explorative nature of Study 1 proved advantageous in observing the impact of the protocol and instruction card on student behaviour. An illustrative anecdote was observed in the explicit group. The protocol stipulates cutting four strips of tape approximately 10 cm in length. Many students obtained a measuring tape to ensure the strips were 10 cm in length. This example highlighted the potential impact of overly detailed instructions on student behaviour.

It should be noted that limitations must be considered. Firstly, the application of a bandage should result in adequate pressure on and around the leg (Nederlandse Vereniging voor Dermatologie en Venereologie (NVDV) & Kenniscentrum Wondzorg, 2015)*.* Determining the ideal pressure for alleviating chronic swelling in the extremities continues to be a topic of contention (Partsch et al., 2011). Nevertheless, a pressure in the range of 40–60 mmHg seems to provide higher efficacy in lower limb lymphedema (Mosti & Cavezzi, 2019)*. *Olszewski (2008) demonstrated that effective lymphatic flow is optimal when bandaging with a pressure of around 40 mmHg. Conversely, it appears that high levels of compression pressure exerted by materials with high stiffness seems to be questionable in lymphedema treatment (Mosti & Cavezzi, 2019)*.* Bandages applied with an initial resting pressure of more than 60 mmHg resulted in a decreasing volume reduction (Partsch et al., 2011). Therefore, the range of 40–60 mmHg was used in this study.

Secondly, attendance at the sessions was not obligatory for the students. Students who were absent from a session were omitted from the study. Consequently, there were fewer students present for the transfer and retention tests. However, this number of students (52) exceeded the number of participants that were included by Masters et al. (2008a), whose design has been used in the test phase. They worked with 36 participants, divided into three groups.

Thirdly, the implicit group from Study 3 was from a different cohort (2022) and it cannot be ruled out that the general level of this cohort was higher. Therefore, the number of European Credit Transfer and Accumulation System (ECTS) at the end of the propaedeutic year for both cohorts was compared. An Independent Samples *t*-test on “ECTS at the end of the propaedeutic year” revealed no significant difference between groups, *t*(296) = 1.17, *p* = 0.24. The mean ECTS credits at the end of the propaedeutic year was 50.34 ECTS (SD = 17.49) for cohort 2021 and 47.90 ECTS (SD = 18.56) for cohort 2022.

Lastly, this study was conducted in a regular classroom setting, which is a less controllable environment than in the setting of an experimental study. In our setting, students from different groups may have shared experiences from their training sessions. Furthermore, we were bound to the existing schedule of training sessions and there may have been some differences between teachers in the application of instructions. Although we invested in calibration sessions, it is possible that some differences in teaching behaviour may have influenced the results. However, the application of an implicit and explicit learning experiment in the more unruly context of our classrooms was precisely the objective of this study. It is questionable whether empirical data can be considered valid when they are only available in a controlled experimental setting and cannot be transferred to real-world settings. Therefore, we consider our experimental design a valuable contribution to the existing literature on implicit and explicit learning*,* both within the context of nursing education and in other educational programmes for healthcare professionals, such as physiotherapy and dermal therapy*.*

## Conclusions

In conclusion, the use of extensive protocols with explicit step-by-step instructions may not be essential for the acquisition of nursing skills, as it is also possible for students to acquire these skills with less instructions. Instead, instructional methods that facilitate implicit learning may be a more beneficial approach, as students in the implicit group demonstrated comparable performance in all studies and tended to perform more consistently when multitasking than students in the explicit group. Further studies will be required to investigate the acquisition of other nursing skills and to demonstrate the clinical effects of educational interventions (Renden & Dikken, 2023; Taraporewalla et al., 2024; Verdoes et al., 2025).

## Electronic supplementary material

Below is the link to the electronic supplementary material.

Supplementary file (DOCX 33 KB)

## Data Availability

Data is provided within the manuscript.

## Dutch protocol with step-by-step instructions (limited to the actions related to bandaging) – See below for English translation

De handeling ACT zwachtelen onder (korte rek):

- Knipt 4 repen pleister af van ongeveer 10 cm en hangt deze binnen handbereik.

Zwachtelen onderbeen.

- Laat zorgvrager been op een steun rusten
- Neemt plaats tegenover zorgvrager op stoel/kruk die op juiste hoogte is gebracht
- Ontbloot het onderbeen
- Brengt buisverband aan
- Knipt anderhalf tot drie keer de lengte van het onderbeen af
  - Trekt buisverband als een kous aan, tot aan knie, laat overtollige verband afhangen over de tenen
  - Polstert indien nodig het onderbeen
- Plaatst voet van zorgvrager in een hoek van 90 graden
- Pakt de eerste zwachtel, begint bij de grote teen en zwachtelt over de voorvoet naar de kleine teen:
  - Zwachtelt van binnen naar buiten
  - Maakt twee windingen om de voorvoet
  - Kijkt in de rol
- Zwachtelt hiel en enkel:
  - Gaat met de volgende slag midden over de voet
  - Maakt een winding op 2/3 van de hiel en 1/3 van de hak
  - Maakt daarna een winding op 1/3 van de hiel en 2/3 van de hak
  - Maakt daarna een winding rond de enkel
- Zwachtelt rest van het onderbeen:
  - Zwachtelt naar boven en volgt de vorm van het been
  - Oefent overal gelijkmatige druk uit
  - Zorgt dat zwachtel glad zit en zonder kreukels (om drukplekken te voorkomen)
  - Maakt vlak onder de knieschijf een circulaire winding (niet te strak)
  - Zwachtelt weer naar beneden tot de zwachtel op is
  - Fixeert de zwachtel met twee pleisters
  - Doet dit op juiste wijze (dus géén stuk pleister rondom het been want dat knelt af)
- Pakt de tweede zwachtel en zwachtelt nog een keer:
- Begint met deze zwachtel weer onderaan bij de tenen
- Zwachtelt tegengesteld aan de eerste zwachtel van buiten naar binnen
- Houdt dezelfde volgorde aan als de eerste zwachtel
- Slaat het afhangende buisverband vanaf de voet terug over het gezwachtelde onderbeen

### English translation of the protocol

The procedure for lower leg bandaging (short stretch):

- Cut four strips of adhesive tape, each approximately 10 cm long, and keep them within easy reach.

Lower Leg Bandaging:

- Ensure the patient’s leg rests on a support
- Sit opposite the patient on a chair or stool adjusted to the correct height
- Expose the lower leg
- Apply tubular bandage
- Cut a piece of tubular bandage measuring 1.5 to 3 times the length of the lower leg
  - Pull the tubular bandage onto the leg like a sock, up to the knee, leaving the excess hanging loosely over the toes
  - Pad the lower leg if necessary
- Position the patient’s foot at a 90-degree angle
- Take the first bandage roll and start at the big toe, wrapping towards the small toe:
  - Wrap from the inner side of the foot to the outer side
  - Make two turns around the forefoot
  - Always look into the roll while wrapping
- Bandage the heel and ankle:
  - Continue with the next turn across the middle of the foot
  - Make a turn covering 2/3 of the heel and 1/3 of the sole
  - Follow with a turn covering 1/3 of the heel and 2/3 of the sole
  - Finish with a turn around the ankle
- Bandage the rest of the lower leg:
  - Wrap upwards, following the contour of the leg
  - Apply even pressure throughout
  - Ensure the bandage is smooth and free of wrinkles to avoid pressure sores
  - Make a circular turn just below the patella (not too tight)
  - Wrap back downwards until the bandage is used up
  - Secure the bandage with two adhesive strips
  - Do this correctly (avoid wrapping the adhesive tape all the way around the leg to prevent constriction)
- Take the second bandage roll and repeat the process:
  - Begin again at the toes, wrapping in the opposite direction (from outer to inner side)
  - Follow the same steps as with the first layer
  - Fold the excess tubular bandage hanging from the foot back over the bandaged lower leg

## Instruction card with minimal instructions—See below for English translation

**Table:**

Doel behaald?	Ja/Nee
Tenen en knie kunnen bewegen	
Zwachtel zakt niet af tijdens lopen	
Geen verkleurde tenen	
Patiënt heeft geen pijn	
Zwachtel sluit aan op het been	
Geen kreukels	
Voet en been zijn bedekt	

### English translation of the instruction card

The figure has been translated from left to right, and the table from top to bottom. Given the use of figurative language, providing a precise translation was challenging. Therefore, we have opted for a contextual rather than a literal translation.

**Objective:** Applying the bandage to the lower leg with the correct pressure.

- Tubular bandage of twice the lower leg length
- Foot at 90°
- Fill cavities with padding
- Create a tube of the leg (apply padding material so that the leg resembles the shape of a tube)
- Look into the roll
- Start at the toes
- Apply tension to the roll
- Two turns around the foot
- Cover the ankle
- Two turns around the heel (2/3 and 1/3)
- Follow the roll’s natural direction from the ankle
- The bandage roll is in the lead
- Take a second bandage roll
- Repeat, start on the other side of the foot

**Objective achieved?** Yes/No.

- Toes and knee can move comfortably
- Bandage does not slip down during walking
- No discoloration of the toes
- Patient experiences no pain
- Bandage is fitted securely
- No wrinkles in the bandage
- Foot and leg are completely covered

## References

[CR1] Beckers, S., Fries, M., Bickenbach, J., Derwall, M., Kuhlen, R., & Rossaint, R. (2005). Minimal instructions improve the performance of laypersons in the use of semiautomatic and automatic external defibrillators. *Critical Care (London, England),**9*(2), R110. 10.1186/cc3033 15774042 10.1186/cc3033 PMC1175919

[CR2] Beckers, S. K., Fries, M., Bickenbach, J., Skorning, M. H., Derwall, M., Kuhlen, R., & Rossaint, R. (2007). Retention of skills in medical students following minimal theoretical instructions on semi and fully automated external defibrillators. *Resuscitation,**72*(3), 444–450. 10.1016/j.resuscitation.2006.08.001 17188417 10.1016/j.resuscitation.2006.08.001

[CR3] Bjørk, I. T., Lomborg, K., Nielsen, C. M., Brynildsen, G., Frederiksen, A. M. S., Larsen, K., Reierson, I. Å., Sommer, I., & Stenholt, B. (2013). From theoretical model to practical use: An example of knowledge translation. *Journal of Advanced Nursing,**69*(10), 2336–2347. 10.1111/JAN.12091 23387968 10.1111/jan.12091

[CR4] Bohn Stafleu van Loghum. (2023). *Skillsonline*. https://skillsonline-bsl.anewspring.nl/do?action=player&type=Scorm&id=4535&courseId=3470

[CR501] Bovill, C., Cook-Sather, A., & Felten, P. (2011). Students as co-creators of teaching approaches, course design, and curricula: Implications for academic developers. *International Journal for Academic Development,**16,* 133–145. 10.1080/1360144X.2011.568690

[CR502] Brooman, S., Darwent, S., & Pimor, A. (2014). The student voice in higher education curriculum design: is there value in listening? *Innovations in Education and Teaching International,**52,* 1–12. 10.1080/14703297.2014.910128

[CR5] Brophy-Williams, N., Driller, M. W., Kitic, C. M., Fell, J. W., & Halson, S. L. (2017). Effect of compression socks worn between repeated maximal running bouts. *International Journal of Sports Physiology and Performance,**12*(5), 621–627. 10.1123/IJSPP.2016-0162 27632195 10.1123/ijspp.2016-0162

[CR6] Button, C., Seifert, L., Chow, J. Y., Araújo, D., & Davids, K. (2020). *Dynamics of skill acquisition: an ecological dynamics approach*. Human Kinetics Publishers.

[CR7] Cabral, D. A. R., Wilson, A. E., & Miller, M. W. (2022). The effect of implicit learning on motor performance under psychological pressure: A systematic review and meta-analysis. *Sport, Exercise, and Performance Psychology,**11*(3), 245–263. 10.1037/SPY0000286

[CR8] Chernikova, O., Heitzmann, N., Stadler, M., Holzberger, D., Seidel, T., & Fischer, F. (2020). Simulation-based learning in higher education: A meta-analysis. *Review of Educational Research,**90*(4), 499–541. 10.3102/0034654320933544

[CR9] Christensen, M. (2011). Advancing nursing practice: Redefining the theoretical and practical integration of knowledge. *Journal of Clinical Nursing,**20*(5–6), 873–881. 10.1111/J.1365-2702.2010.03392.X 21320209 10.1111/j.1365-2702.2010.03392.x

[CR10] Cohen, J. (1988). *Statistical power analysis for the behavioral sciences* (2nd ed.). Lawrence Erlbaum.

[CR11] Crowe, S., Ewart, L., & Derman, S. (2018). The impact of simulation based education on nursing confidence, knowledge and patient outcomes on general medicine units. *Nurse Education in Practice,**29*, 70–75. 10.1016/J.NEPR.2017.11.017 29190590 10.1016/j.nepr.2017.11.017

[CR12] Damstra, R. J., Brouwer, E. R., & Partsch, H. (2008). Controlled, comparative study of relation between volume changes and interface pressure under short-stretch bandages in leg lymphedema patients. *Dermatologic Surgery Official Publication for American Society for Dermatologic Surgery,**34*(6), 773–779. 10.1111/J.1524-4725.2008.34145.X 10.1111/j.1524-4725.2008.34145.x 18336577

[CR13] Damstra, R. J., & Partsch, H. (2009). Compression therapy in breast cancer-related lymphedema: A randomized, controlled comparative study of relation between volume and interface pressure changes. *Journal of Vascular Surgery,**49*(5), 1256–1263. 10.1016/J.JVS.2008.12.018 19394553 10.1016/j.jvs.2008.12.018

[CR14] Davids, K., Button, C., & Bennett, S. (2008). *Dynamics of skill acquisition: A constraints-led approach*. Human Kinetics Publishers.

[CR15] Dikken, J., Bakker, A., Hoogerduijn, J. G., & Schuurmans, M. J. (2018). Comparisons of knowledge of Dutch nursing students and hospital nurses on aging. *Journal of Continuing Education in Nursing,**49*(2), 84–90. 10.3928/00220124-20180116-08 29381172 10.3928/00220124-20180116-08

[CR16] El-Kishawi, M., Khalaf, K., Murray, C., Odeh, R., & Winning, T. (2023). Impact of guidance and multitasking on manual dexterity skills in dentistry. *European Journal of Dentistry,**17*(2), 387–397. 10.1055/S-0042-1743155 35436786 10.1055/s-0042-1743155 PMC10329529

[CR17] Ericsson, K. A. (2014). The road to excellence: The acquisition of expert performance in the arts and sciences, sports, and games. In K. A. Ericsson (Ed.), *The road to excellence: The acquisition of expert performance in the arts and sciences, sports, and games. *Lawrence Erlbauw Associates.

[CR18] Fitts, P. M., & Posner, M. I. (1967). *Learning and skilled performance in human performance*. Brooks Cole.

[CR19] Günay, U., & Kılınç, G. (2018). The transfer of theoretical knowledge to clinical practice by nursing students and the difficulties they experience: A qualitative study. *Nurse Education Today,**65*, 81–86. 10.1016/J.NEDT.2018.02.031 29547811 10.1016/j.nedt.2018.02.031

[CR20] Hawkins, N., Jeong, S., & Smith, T. (2019). Coming ready or not! An integrative review examining new graduate nurses’ transition in acute care. *International Journal of Nursing Practice*. 10.1111/ijn.12714 10.1111/ijn.12714 30537440

[CR21] Huang, H.-J., & Mercer, V. S. (2001). Dual-task methodology: Applications in studies of cognitive and motor performance in adults and children. *Pediatric Physical Therapy,**13*(3), 133–140. 10.1097/00001577-200110000-00005 17053670

[CR22] Hutter, R. I., Renden, P. G., Kok, M., Oudejans, R., Koedijk, M., & Kleygrewe, L. (2023). Criteria for the high quality training of police officers. *Police Conflict Management,**II*, 7–32. 10.1007/978-3-031-41100-7_2

[CR23] Kahol, K., Vankipuram, M., Patel, V. L., & Smith, M. L. (2011). Deviations from protocol in a complex Trauma environment: Errors or innovations? *Journal of Biomedical Informatics,**44*(3), 425–431. 10.1016/J.JBI.2011.04.003 21496496 10.1016/j.jbi.2011.04.003

[CR24] Kal, E., Prosée, R., Winters, M., & Van Der Kamp, J. (2018). Does implicit motor learning lead to greater automatization of motor skills compared to explicit motor learning? A systematic review. *PLoS ONE,**13*(9), e0203591. 10.1371/JOURNAL.PONE.0203591 30183763 10.1371/journal.pone.0203591 PMC6124806

[CR25] Kleynen, M., Braun, S. M., Bleijlevens, M. H., Lexis, M. A., Rasquin, S. M., Halfens, J., Wilson, M. R., Beurskens, A. J., & Masters, R. S. W. (2014). Using a delphi technique to seek consensus regarding definitions, descriptions and classification of terms related to implicit and explicit forms of motor learning. *PLoS ONE,**9*(6), e100227. 10.1371/JOURNAL.PONE.0100227 24968228 10.1371/journal.pone.0100227 PMC4072669

[CR26] Kleynen, M., Braun, S. M., Rasquin, S. M. C., Bleijlevens, M. H. C., Lexis, M. A. S., Halfens, J., Wilson, M. R., Masters, R. S. W., & Beurskens, A. J. (2015). Multidisciplinary views on applying explicit and implicit motor learning in practice: An international survey. *PLoS ONE,**10*(8), e0135522. 10.1371/JOURNAL.PONE.0135522 26296203 10.1371/journal.pone.0135522 PMC4546413

[CR27] Kok, M. J. (2023). *Motor Learning Method Matters in Physical Education* [PhD-Thesis - Research and graduation internal, Vrije Universiteit Amsterdam]. 10.5463/thesis.221

[CR28] Lam, W. K., Maxwell, J. P., & Masters, R. S. W. (2009). Analogy versus explicit learning of a modified basketball shooting task: Performance and kinematic outcomes. *Journal of Sports Sciences,**27*(2), 179–191. 10.1080/02640410802448764 19153868 10.1080/02640410802448764

[CR29] Liao, C. M., & Masters, R. S. W. (2001). Analogy learning: A means to implicit motor learning. *Journal of Sports Sciences,**19*(5), 307–319. 10.1080/02640410152006081 11354610 10.1080/02640410152006081

[CR30] Lohse, K. R., Sherwood, D. E., & Healy, A. F. (2010). How changing the focus of attention affects performance, kinematics, and electromyography in dart throwing. *Human Movement Science,**29*(4), 542–555. 10.1016/J.HUMOV.2010.05.001 20541275 10.1016/j.humov.2010.05.001

[CR31] Marton, F. (2006). Sameness and difference in transfer. *Journal of the Learning Sciences,**15*(4), 499–535. 10.1207/s15327809jls1504_3

[CR32] Masters, R., Lo, C. Y., Maxwell, J. P., & Patil, N. G. (2008a). Implicit motor learning in surgery: Implications for multi-tasking. *Surgery,**143*(1), 140–145. 10.1016/j.surg.2007.06.018 18154942 10.1016/j.surg.2007.06.018

[CR33] Masters, R. S. W., Poolton, J. M., & Maxwell, J. P. (2008b). Stable implicit motor processes despite aerobic locomotor fatigue. *Consciousness and Cognition,**17*(1), 335–338. 10.1016/J.CONCOG.2007.03.009 17470398 10.1016/j.concog.2007.03.009

[CR34] Masters, R. S. W., Poolton, J. M., Maxwell, J. P., & Raab, M. (2010). Implicit motor learning and complex decision making in time-constrained environments. *Journal of Motor Behavior,**40*(1), 71–79. 10.3200/JMBR.40.1.71-80 10.3200/JMBR.40.1.71-80 18316298

[CR35] Masters, R. S. W., van Duijn, T., & Uiga, L. (2019). Advances in implicit motor learning. *Skill Acquisition in Sport. *Routledge. 10.4324/9781351189750-5

[CR36] Mosti, G., & Cavezzi, A. (2019). Compression therapy in lymphedema: Between past and recent scientific data. *Phlebology: The Journal of Venous Disease,**34*(8), 515–522. 10.1177/0268355518824524 10.1177/0268355518824524 30626269

[CR37] Nederlandse Vereniging voor Dermatologie en Venereologie (NVDV), & Kenniscentrum Wondzorg. (2015). *Compressietherapie aan de onderste extremiteiten*.

[CR38] Olszewski, W. L. (2008). Contractility patterns of human leg lymphatics in various stages of obstructive lymphedema. *Annals of the New York Academy of Sciences,**1131*(1), 110–118. 10.1196/ANNALS.1413.010 18519964 10.1196/annals.1413.010

[CR39] Partsch, H., Clark, M., Bassez, S., Benigni, J. P., Becker, F., Blazek, V., Caprini, J., Cornu-Thénard, A., Hafner, J., Flour, M., Jünger, M., Moffatt, C., & Neumann, M. (2006). Measurement of lower leg compression in vivo: Recommendations for the performance of measurements of interface pressure and stiffness: Consensus statement. *Dermatologic Surgery,**32*(2), 224–233. 10.1111/J.1524-4725.2006.32039.X 16442043 10.1111/j.1524-4725.2006.32039.x

[CR40] Partsch, H., Damstra, R. J., & Mosti, G. (2011). Dose finding for an optimal compression pressure to reduce chronic edema of the extremities. *International Angiology: A Journal of the International Union of Angiology,**30*(6), 527–533. 22233613

[CR41] Poolton, J. M., Masters, R. S. W., & Maxwell, J. P. (2007). Passing thoughts on the evolutionary stability of implicit motor behaviour: Performance retention under physiological fatigue. *Consciousness and Cognition,**16*(2), 456–468. 10.1016/j.concog.2006.06.008 16876433 10.1016/j.concog.2006.06.008

[CR42] Poolton, J. M., Zhu, F. F., Malhotra, N., Leung, G. K. K., Fan, J. K. M., & Masters, R. S. W. (2016). Multitask training promotes automaticity of a fundamental laparoscopic skill without compromising the rate of skill learning. *Surgical Endoscopy,**30*(9), 4011–4018. 10.1007/S00464-015-4713-9 26743112 10.1007/s00464-015-4713-9 PMC4992021

[CR43] Renden, P. G., & Dikken, J. (2023). Introducing the constraints-led approach in nurse education: An innovative perspective on skill acquisition. *Nurse Education Today,**121*, 105672. 10.1016/J.NEDT.2022.105672 36502661 10.1016/j.nedt.2022.105672

[CR44] Taraporewalla, K., Eriksson, L., Barach, P., Lipman, J., & Zundert, A. (2024). Approaches to teaching medical procedural skills: A scoping review. *Medical Research Archives*. 10.18103/mra.v12i5.5394

[CR45] Taraporewalla, K., van Zundert, A., Watson, M., & Renshaw, I. (2022). The ecological-dynamics framework for medical skills. *Healthcare,**11*(1), 38. 10.3390/healthcare11010038 36611498 10.3390/healthcare11010038 PMC9819195

[CR46] Van Duijn, T., Hoskens, M. C. J., & Masters, R. S. W. (2019). Analogy instructions promote efficiency of cognitive processes during hockey push-pass performance. *Sport, Exercise, and Performance Psychology,**8*(1), 7–20. 10.1037/SPY0000142

[CR47] Van Merriënboer, J. G., Kirschner, P., & Frèrejean, J. (2025). *Ten steps to complex learning: A systematic approach to&nbsp;four-component instructional design*. Fourth edn., Routledge.

[CR500] Verdoes, L., Dikken, J., Van Brakel-Van Lobenstein, N., Van Leeuwen-Prins, S.T., De Waal, G.M., & Renden, P.G. (2025). Embracing a constraints-led approach for skills acquisition in nursing education: Insights from a focus group study. *International Journal of Educational Research Open,**9*, 100459. 10.1016/j.ijedro.2025.100459.

