# Supplementary material for: Teaching nursing skills without detailed protocols: effects of an implicit learning strategy in nursing education
Source: Adv Health Sci Educ Theory Pract. 2025 Mar 25;30(5):1541–66. doi: 10.1007/s10459-025-10421-y (PMC12572041; doi:10.1007/s10459-025-10421-y)
Supplement: Supplementary file 1 — Supplementary file (DOCX 33 KB) [file 10459_2025_10421_MOESM1_ESM.docx]

**Supplementary files**

**Supplementary files Table 1.** Mean ± standard deviation of dependent variables of Study 1

|  | **Explicit** | | **Implicit** | |
| --- | --- | --- | --- | --- |
|  | Attempt 1 | Attempt 2 | Attempt 1 | Attempt 2 |
| *Recall* |  |  |  |  |
| Movement-related rules |  | 6.53 (3.13) |  | 7.41 (2.56) |
| *Performance* |  |  |  |  |
| Toes and knee can move (1-5)* | 4.67 (.69) | 4.67 (.49) | 4.78 (.42) | 4.74 (.45) |
| Bandage does not slip when walking (1-5)* | 5.00 (.00) | 5*.*00 (.00) | 4*.*96 (.21) | 5*.*00 (.00) |
| No discolored toes (1-5)* | 4.94 (.25) | 5.00 (.00) | 4.95 (.21) | 5.00 (.00) |
| Patient has no pain (1-5)* | 4.83 (.38) | 5.00 (.00) | 4.87 (.34) | 4.83 (.39) |
| Bandage fits tightly around the leg (1-5)* | 4.11 (.96) | 4.28 (.58) | 3.74 (1.01) | 4.09 (.67) |
| No wrinkles (1-5)* | 3.28 (.67) | 4.17 (.79) | 3.35 (.83) | 4.43 (.67) |
| Foot and leg are covered (1-5)* | 4.78 (.55) | 4.89 (.32) | 4.96 (.21) | 4.87 (.34) |
| *Time* |  |  |  |  |
| Time practicing (seconds) | 759.26 (179.79) | 601.32 (177.70) | 794.57 (214.27) | 648.52 (148.08) |
| *Reading instructions* |  |  |  |  |
| Frequency looking at instructions Phase 1 | 3.78 (4.11) | .22 (.73) | 1.78 (1.91) | .17 (.49) |
| Frequency looking at instructions Phase 2 | 2.67 (3.68) | .22 (.73) | 1.48 (1.38) | .13 (.34) |
| Frequency looking at instructions Phase 3 | 1.17 (2.62) | .00 (.00) | .22 (.518) | .04 (.21) |
| Time looking at instructions Phase 1 (seconds) | 34.06 (38.78) | 1.22 (4.71) | 4.43 (6.17) | .39 (1.12) |
| Time looking at instructions Phase 2 (seconds) | 19.56 (24.84) | 1.56 (4.97) | 4.35 (5.85) | .43 (.99) |
| Time looking at instructions Phase 3 (seconds) | 5.94 (12.89) | .00 (.00) | .26 (.62) | .04 (.21) |

* Criteria were rated 1 (wrong) – 5 (correct)

**Supplementary files Table 2.** Statistical analyses of Study 1

|  | Group | Attempt | Group*Attempt |
| --- | --- | --- | --- |
| *Recall* |  |  |  |
| Movement-related rules | *t*(37*) =* .070*, p* = .34 |  |  |
| *Performance* |  |  |  |
| Toes and knee can move | *F*(1.39) = .44,  *p* = .51 | *F*(1.39) = .08,  *p* = .78 | *F*(1.39) = .08,  *p* = .78 |
| Bandage does not slip when walking | *F*(1.39) = .78,  *p* = .38 | *F*(1.39) = .78,  *p* = .38 | *F*(1.39) = .78,  *p* = .38 |
| No discoloured toes | *F*(1.36) = .05,  *p* = .82 | *F*(1.36) = 2.05,  *p* = .16 | *F*(1.36) = .05,  *p* = .82 |
| Patient has no pain | *F*(1.39) = .80,  *p* = .38 | *F*(1.39) = .80,  *p* = .38 | *F*(1.39) = 2.33,  *p* = .14 |
| Bandage fits tightly around the leg | *F*(1.39) = 1.74,  *p* = .20 | *F*(1.39) = 2.92,  *p* = .96 | *F*(1.39) = .36,  *p* = .55 |
| No wrinkles | *F*(1.39) = .70,  *p* = .41 | *F*(1.39) = 71.16,  *p* = < .001, *η^2^* = .65 | *F*(1.39) = .72,  *p* = .40 |
| Foot and leg are covered | *F*(1.39) = .65,  *p* = .42 | *F*(1.39) = .04,  *p* = .84 | *F*(1.39) = 2.76,  *p* = .11 |
| *Time* |  |  |  |
| Time practicing | *F*(1.39) = .79,  *p* = .38 | *F*(1.39) = 22,37,  *p* < .001, *η^2^* = .36 | *F*(1.39) = .03,  *p* = .85 |
| *Reading instructions* |  |  |  |
| Frequency looking at instructions Phase 1 | *F*(1.39) = 3.49,  *p* = 0.07 | *F*(1.39) = 35.98,  *p* < .001, *η^2^* = .48 | *F*(1.39) = 5.11,  *p* < .05, *η^2^* = .12 |
| Frequency looking at instructions Phase 2 | *F*(1.39) = 2.09,  *p* = .16 | *F*(1.39) = 21.98,  *p* < 0.001, *η^2^* = .36 | *F*(1.39) = 1.84,  *p* = .18 |
| Frequency looking at instructions Phase 3 | *F*(1.39) = 2.59,  *p* = .12 | *F*(1.39) = 5.81,  *p* < .05, *η^2^* = .13 | *F*(1.39) = 3.19,  *p* < .08 |
| Time looking at instructions Phase 1 (seconds) | *F*(1.39) = 12.58,  *p* < .05, *η^2^* = .24 | *F*(1.39) = 21.79,  *p* < .001, *η^2^* = .36 | *F*(1.39) = 13.28,  *p* < .001, *η^2^* = .25 |
| Time looking at instructions Phase 2 (seconds) | *F*(1.39) = 8.06,  *p* < .05, *η^2^* = .17 | *F*(1.39) = 18.30,  *p* < .001, *η^2^* = .32 | *F*(1.39) = 7.56,  *p* < .05, *η^2^* = .16 |
| Time looking at instructions Phase 3 (seconds) | *F*(1.39) = 4.42,  *p* < .05, *η^2^* = .10 | *F*(1.39) = 5.28,  *p* < .05, *η^2^* = .12 | *F*(1.39) = 4.56,  *p* < .05, *η^2^* = .11 |

**Supplementary files Table 3.** Mean ± standard deviation of dependent variables of Study 2

|  | **Explicit (*n* = 27)** | | | **Implicit (*n* = 25)** | | |
| --- | --- | --- | --- | --- | --- | --- |
|  | Retention test | Transfer test | Both tests | Retention test | Transfer test | Both tests |
| Movement-related rules |  |  | 15.43 (6.09) |  |  | 11.65 (3.23) |
| Pressure of the bandage (mmHg) | 44.48 (15.65) | 49.93 (17.11) |  | 44.36 (16.05) | 48.12 (14.10) |  |
| Consistency pressure *(*mmHg) |  |  | 13.37 (11.77) |  |  | 12.56 (7.68) |
| Deviation from range 40-60 *(*mmHg) | 5.37 (7.99) | 5.93 (7.35) |  | 5.96 (7.08) | 3.88 (6.36) |  |
| Pressure between 40-60 mmHg (yes) | 14 (52%) | 11 (41%) | 6 (22%) | 11 (44%) | 15 (60%) | 7 (28%) |
| Performance time (seconds) | 395.00 (100.57) | 346.44 (112.96) |  | 381.16 (90.28) | 358.20 (92.19) |  |
| Consistency time (seconds) |  |  | 79.52 (73.45) |  |  | 67.76 (57.18) |

**Supplementary files Table 4.** Statistical analyses of Study 2

|  | Group | Test | Group*Test |
| --- | --- | --- | --- |
| Movement-related rules | *t*(44) = 2.63, *p* = < .05, *d* = .78 |  |  |
| Pressure of the bandage | *F*(50) = .065, *p* = .80 | *F*(50) = 4.35, *p* < .05, *η^2^* = .80 | *F*(50) = .15, *p* = .70 |
| Consistency pressure | *t*(50) = .30, *p* = .77 |  |  |
| Deviation from the range 40-60 mmHg | *F*(50) = .23, *p* = .64 | *F*(*F*(50) = .35, *p* = .56 | *F*(50) = 1.03, *p* = .32 |
| Pressure between 40-60 mmHg in retention test | *X^2^*(1) = .32, *p* = .57 |  |  |
| Pressure between 40-60 mmHg in transfer test | *X^2^*(1) = 1.93, *p* = .17 |  |  |
| Pressure between 40-60 mmHg in both tests | *X^2^*(1) = .23, *p* = .63 |  |  |
| Performance time | *F*(50) = .00, *p* = .97 | *F*(50) = 7.77, *p* < .05, *η^2^* = .13 | *F*(50) = .99, *p* = .32 |
| Consistency time | *t*(50) = .64, *p* = .53 |  |  |

**Supplementary files Table 5.** Mean ± standard deviation of dependent variables of Study 3

|  | **Explicit (*n* = 27)** | | | **Implicit (*n* = 27)** | | |
| --- | --- | --- | --- | --- | --- | --- |
|  | Retention test | Transfer test | Both tests | Retention test | Transfer test | Both tests |
| Movement-related rules |  |  | 15.43 (6.09) |  |  | 11.42 (3.59) |
| Pressure of the bandage (mmHg) | 44.48 (15.65) | 49.93 (17.11) |  | 41.81 (10.36) | 44.56 (12.09) |  |
| Consistency pressure *(*mmHg) |  |  | 13.37 (11.77) |  |  | 7.85 (6.73) |
| Deviation from range 40-60 *(*mmHg) | 5.37 (7.99) | 5.93 (7.35) |  | 3.26 (5.76) | 3.22 (5.85) |  |
| Pressure between 40-60 mmHg (yes) | 14 (52%) | 11 (41 %) | 6 (22%) | 18 (67%) | 19 (70%) | 15 (56%) |
| Performance time (seconds) | 395 (100.57) | 346.44 (112.96) |  | 375.15 (111.58) | 341.81 (115.92) |  |
| Consistency time (seconds) |  |  | 79.52 (73.45) |  |  | 57.63 (53.45) |

**Supplementary files Table 6.** Statistical analyses of Study 3

|  | Group | Test | Group*Test |
| --- | --- | --- | --- |
| Movement-related rules | *t*(52) = 2.115, *p* < .05, *d* = .80 |  |  |
| Pressure of the bandage | *F*(52) = 1,47, *p* = .231 | *F*(52) = 4.58, *p* < .05, *η^2^* = .08 | *F*(52) = .50, *p* = .48 |
| Consistency pressure | *t*(52) = 2.12, *p* < .05, *d =* .58 |  |  |
| Deviation outside the range 40-60 mmHg | *F*(52) = 2.85, *p* = .10 | *F*(52) = .05, *p* = .83 | *F*(52) = .06, *p* = .80 |
| Pressure between 40-60 mmHg in retention test | *χ²*(1) = 1.23, *p* = .27 |  |  |
| Pressure between 40-60 mmHg in transfer test | *χ²*(1) = 4.80, *p* <.05. |  |  |
| Pressure between 40-60 mmHg in both tests | *χ²*(1) = 6.31, *p* < .05 |  |  |
| Performance time | *F*(52) = .20, *p* = .66 | *F*(52) = 12.36, *p* < .001, *η^2^* = .19 | *F*(52) = .43, *p* = .52 |
| Consistency time | *t*(52) = 2.13, *p* = .22 |  |  |
